# *NucleoFind*: a deep-learning network for interpreting nucleic acid electron density

**DOI:** 10.1093/nar/gkae715

**Published:** 2024-08-20

**Authors:** Jordan S Dialpuri, Jon Agirre, Kathryn D Cowtan, Paul S Bond

**Affiliations:** York Structural Biology Laboratory, Department of Chemistry, University of York, York, UK; York Structural Biology Laboratory, Department of Chemistry, University of York, York, UK; York Structural Biology Laboratory, Department of Chemistry, University of York, York, UK; York Structural Biology Laboratory, Department of Chemistry, University of York, York, UK

## Abstract

Nucleic acid electron density interpretation after phasing by molecular replacement or other methods remains a difficult problem for computer programs to deal with. Programs tend to rely on time-consuming and computationally exhaustive searches to recognise characteristic features. We present *NucleoFind*, a deep-learning-based approach to interpreting and segmenting electron density. Using an electron density map from X-ray crystallography obtained after molecular replacement, the positions of the phosphate group, sugar ring and nitrogenous base group can be predicted with high accuracy. On average, 78% of phosphate atoms, 85% of sugar atoms and 83% of base atoms are positioned in predicted density after giving *NucleoFind* maps produced following successful molecular replacement. *NucleoFind* can use the wealth of context these predicted maps provide to build more accurate and complete nucleic acid models automatically.

## Graphical Abstract

**Graphical Abstract:**
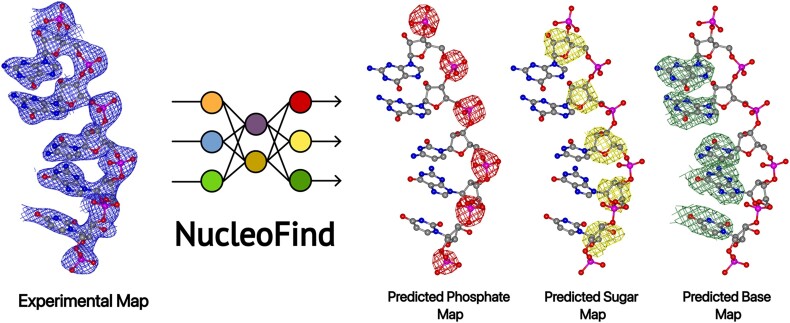

## Introduction

Interpretation of macromolecular electron density maps from X-ray crystallography can be trivial for an experienced crystallographer but is a conceptually difficult problem for computer algorithms to solve. Despite this, many software packages have been successful at automatically building macromolecular structures into electron density. In protein atomic model building, algorithmic approaches that rely on orientation-dependent likelihood functions (1) or free atom placement (2) are mature and work well. However, the electron density of nucleic acid-containing structures is often more difficult to interpret than that of proteins, especially after obtaining phase estimates by protein molecular replacement. Nevertheless, automated model building can work well in some cases after molecular replacement, but many still require further manual attention (3).

The technical challenge for a program to understand complex 3D shapes with high variability between instances fits well with the abilities of deep-learning-based methods. In this work, we present a set of deep-learning networks for the interpretation and segmentation of electron density maps that originate from structures containing nucleic acids. The networks can positively identify the three constituent parts of a nucleotide, the phosphate group, the ribose sugar and the nitrogenous base before an atomic model is built. The predictions obtained from these networks are beneficial when attempting to build nucleic acid features into electron density following molecular replacement. The context obtained from the predictions has been used to enhance the capabilities of automated model-building software in historically difficult cases, such as when building large protein-nucleic acid complexes after molecular replacement using a protein template.

### Background

The neural network architecture at the core of this software package is based upon the U-Net architecture (4). The U-Net is a convolutional neural network that was created to analyse and segment two-dimensional biomedical images, with a strong focus on utilising the relatively limited number of data samples in an efficient and effective way. As opposed to taking in the entire data sample at once, the U-Net instead relies on a small portion or ‘chunk’ of data being supplied to the network for analysis, the results of which are then combined to classify the entire data sample. In the original U-Net deployment, this data was two-dimensional images, but further research expanded the U-Net to the third dimension for use in other biomedical areas (5). The original 3D U-Net implementation used a collection of 2D images to generate a three-dimensional dataset, however, other 3D datasets can be used too, such as a crystallographic electron density map.

This type of convolutional neural network can also be described as an encoder-decoder network with opposing downsampling and upsampling portions. Similar network architectures have already been shown to be extremely useful for characterising experimental data in structural biology. *Haruspex* (6) demonstrated the impressive utility of these convolutional neural networks by annotating the secondary structure of cryo-electron microscopy (cryo-EM) density maps. The network at the core of *Haruspex* assigned a probability of each point in the density map corresponding to an α-helix, β-sheet, nucleotide, or an unassigned feature. The network received cubes of density between 40^3^ Å^3^ and 48^3^ Å^3^ in volume as input, which allowed for sufficient secondary structure coverage while also minimising model complexity. This annotation proved useful in informing downstream automated model-building pipelines.

Since this, approaches which replace the classical algorithmic model-building software pipelines with methods more dependent on neural networks have emerged. *DeepTracer* (7) uses four separate encoder-decoder (U-Net) networks to obtain precise structural information from cryo-EM density alone. The first network categorises each point in the map as belonging to specific atom types, while the second analyses each point for its proximity to the protein backbone. In a similar way to the network in *Haruspex*, a third U-Net network classifies each point by its secondary structure, and the final network assigns each point to an amino acid type. Combining the outputs of each of these classifications, in particular, the protein backbone and atom-type networks allows for efficient chain tracing. Using more classical optimisation algorithms from the atomic positions and the other classification networks, *DeepTracer* is able to automatically build models into cryo-EM density maps quickly.

A similar network, the modified feature pyramid is present in the popular software package *ModelAngelo*. This encoder-decoder network characterises each point in a cryo-EM density map as either the α-carbon atom in a protein or the phosphorous atom in a nucleic acid in a similar way to the atom-type network in *DeepTracer*. However, while *DeepTracer* relies on classical algorithmic model-building methods to transform the network classifications into accurate protein models, *ModelAngelo* achieves this with a further graph neural network (GNN). This GNN optimises the positions of the located residues using information from the map, the sequence and the geometry between neighbours. A big advantage of *ModelAngelo* over *DeepTracer* for model building is the ability to build nucleic acids in addition to protein features.

Recently, another program capable of building nucleic acid structures, *CryoREAD* was released (8). Again, the U-Net architecture was used to identify and classify parts of the cryo-EM density. In this case, the networks classified each point in the map as sugar, phosphate, base or none. Following this, classical chain tracing and sequence assignment produces an accurate full-atom model. The utility of these convolutional neural networks in cryo-EM is clear, but their applicability has also been shown with protein crystallographic density maps. Using a 3D fully-convolutional DenseNet, which has similar downsampling and upsampling stages to a basic U-Net, Godó *et al.* (9) segmented crystallographic protein density maps into each residue type without requiring sequence information.

Using a similar network architecture, *NucleoFind* aims to identify the constituent parts of nucleic acid electron density from X-ray crystallography, both for nucleic acid-only structures and protein-nucleic acid complexes, an area which is currently unexplored. *NucleoFind* outputs these predicted maps for each nucleic acid group type as a CCP4 map file. These predicted maps are then used to guide fast automated model building, but can also be used to aid interactive model building.

## Materials and methods

### Neural network architecture

**Figure 1.**
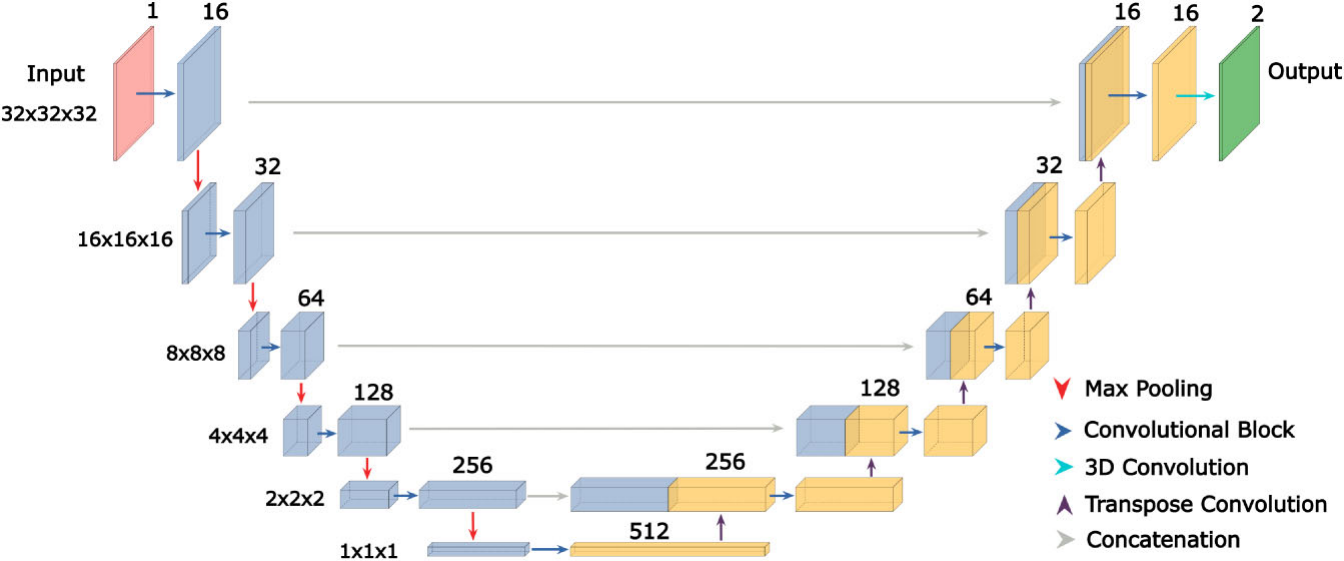
Schematic view of the 3D U-Net architecture. The encoder-decoder network first downsamples the data of shape (32, 32, 32, 1) to a vector form of shape (1, 1, 1, 512). The vector is then upsampled back to an output of shape (32, 32, 32, 2), where the two output channels represent the probability of the target group being, or not being, at each point in the output box.

The network created for nucleic acid semantic segmentation is based on the 3D U-Net with slight alterations to the normalisation functions, shown in Figure 1. The input to the network is a cube with three spatial dimensions of length 32 and one filter dimension of length 1. In crystallographic terms, the spatial dimensions represent a cubic grid of 32 points in each dimension with 0.7 Å grid spacing, with the density value at each point in the filter dimension. Following the input layer is a set of downsampling blocks which encompass a range of operations to modify the incoming data to half the number of spatial dimensions and twice the number of filter dimensions. Given an input data set with shape (*n*, *n*, *n*, *m*), a downsampling block will transform and return a dataset with shape ($\frac{n}{2}$, $\frac{n}{2}$, $\frac{n}{2}$, 2*m*). The upsampling blocks operate oppositely, taking in a (*n*, *n*, *n*, *m*) input and returning a (2*n*, 2*n*, 2*n*, $\frac{m}{2}$) output.

#### Downsampling

In the downsampling portion of the model, the inputted electron density of shape (32, 32, 32, 1) is transformed into a vector representation of shape (1, 1, 1, 512). This is achieved through a series of downsampling blocks, the first of which changes the number of filters of the inputted data from 1 to 16. All subsequent downsampling blocks simply double the number of values in the filter dimension and halve the spatial dimensions. Contained within each of these downsampling blocks are two convolutional blocks followed by a max-pooling operation. Each convolutional block comprises a convolutional operation, instance normalisation operation (10) and rectified linear unit activation operation. For each of the convolutions, padding was applied so the output was the same size as the input, all parameters used in these operations are shown in Supplementary Information Section 1.1.

#### Bottleneck

The vector representation which is the result of the downsampling portion of the network is then further processed during the bottleneck portion of the network. This part of the network should theoretically contain almost all of the information required for the network to segment the input map. In the bottleneck, two convolutional blocks are applied with normalisation operations removed to prevent distortion of the critical vector representation.

#### Upsampling

**Figure 2.**
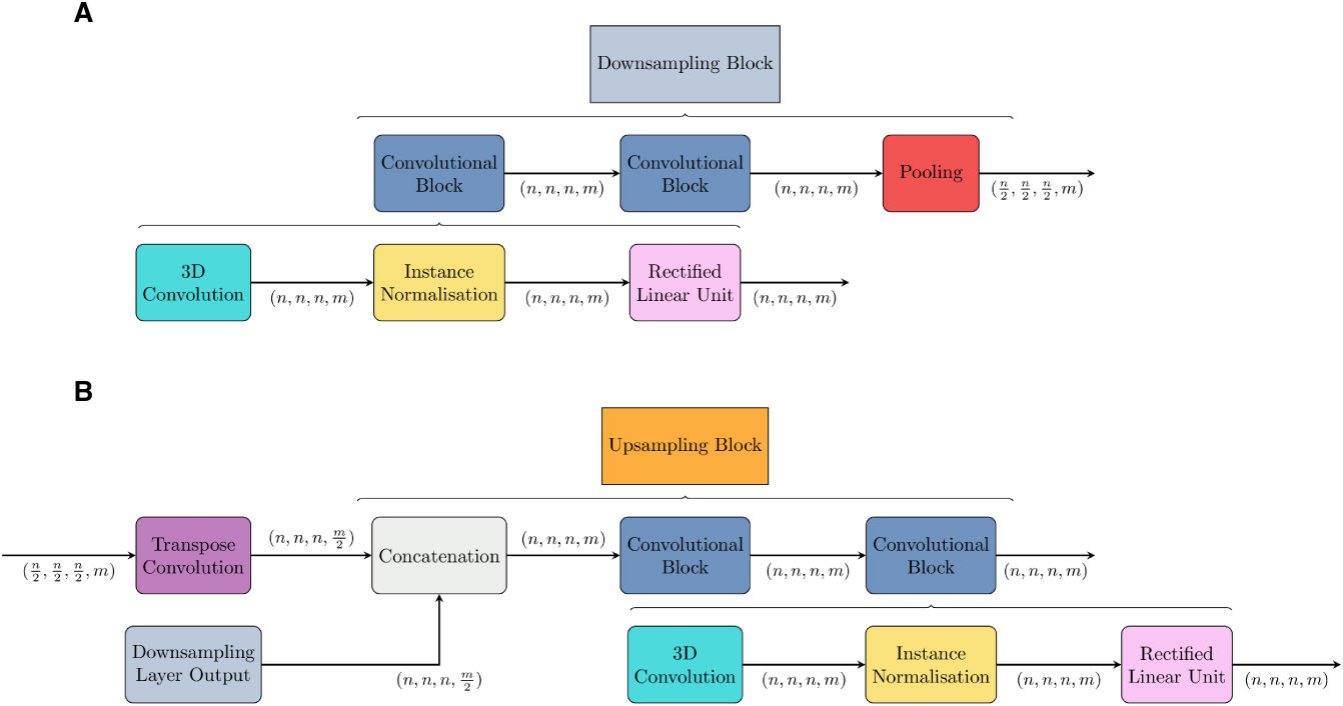
(**A**) Schematic representation of the downsampling block which takes in a tensor of shape (*n*, *n*, *n*, *m*) and downsamples it to a tensor of shape $(\frac{n}{2}, \frac{n}{2}, \frac{n}{2}, 2m)$. Each downsampling block contains two convolutional blocks followed by a single pooling operation. (**B**) Schematic representation of the upsampling block which takes in a tensor of shape (*n*, *n*, *n*, *m*) and converts it into a tensor of shape ($2n, 2n, 2n, \frac{m}{2}$). Each upsampling block contains a transpose convolution doubles the inputted spatial dimensions, followed by a concatenation operation which combines the filter dimensions of the corresponding downsampling operation with the filter dimension of the transpose convolution. Following this, are two standard convolutional blocks.

From the vector representation, the network upsamples the data, such that the output tensor has the same spatial dimensions as the input tensor. This is completed using five upsampling blocks. Each upsampling block consists of a transposed convolution which doubles the spatial dimensions and halves the filter dimensions. This result is then concatenated with the corresponding (same spatial dimensions) output from the downsampling block and two final convolutional and normalisation operations are applied which maintain both spatial and filter dimensionality. This is shown schematically in Figure 2B.

#### Output

Once the network has reached the final upsampling block, a final convolution with softmax activation is applied to transform the filter dimension from 16 to 2 outputs at each of the spatial points. The two outputs represent the classification at each point, i.e. the probability that this point is and is not within 1.5 Å of the target group.

Overall, the network architecture described is very similar to that used in *Haruspex*, *DeepTracer* and *CryoREAD* with only slight changes to the input size, number of layers and filters, and the normalisation layers used throughout the networks.

### Training

#### Dataset creation

The dataset used for the network training originated from the Protein Data Bank (PDB) (11). All structures used were obtained using X-ray diffraction, with no resolution filter applied. From this collection of nucleic acid-containing structures in the PDB, 1000 protein-nucleic acid structures were reserved for use in later unseen testing. In total, the starting dataset contained 2711 structures containing only nucleic acid and 8369 structures containing both protein and nucleic acid polymers. Of the 2711 nucleic acid-only structures, 1558 were DNA, 1119 were RNA and 34 were DNA/RNA complexes. Of the 8369 protein–nucleic acid structures, 5754 were protein–DNA complexes, 2182 were protein–RNA complexes and 433 were protein–DNA/RNA complexes.

Maps to interpret were taken from the RCSB (12) using phases from the final deposited structure, calculated by the RCSB using *DCC* (13). To supplement the dataset with more realistic model-building cases, the maps of all protein and nucleic acid-containing models were recalculated to better resemble the output of molecular replacement. To achieve this, all non-protein molecules were removed, and the *B*-factors for the remaining protein residues were set to the average *B*-factor value of the remaining model. This model was then refined with *REFMAC5* (14) to obtain a more realistically phased map. This map in MTZ form and the deposited model were then added to the dataset. Histograms showing the distribution of resolutions in the training data are shown in Supplementary Figures S1 and S2.

#### Dataset preprocessing

As the neural network receives a cube of electron density as input, it is important that the grid spacing is consistent throughout all of the training datasets. To achieve this, a section of map containing the asymmetric unit was first interpolated onto a regular orthogonal grid with a spacing of 0.7 Å. From this, a map for each target group (phosphate, sugar and base) was generated by labelling areas of density within 1.5 Å of a target atom in the deposited model. These maps represent the desired output that the predictive networks are aiming for. For each MTZ file from the dataset, four maps were output, the original interpolated map and three target maps, all in CCP4 map file format.

#### Training scheme

Dataset preprocessing yields maps originating from three sources: nucleic acid-only structures, protein-nucleic acid structures with deposited phases and protein-nucleic acid structures with post-molecular replacement-like maps. During training, these three map sources were cycled continuously to aid the network in learning information about realistic examples. At each iteration in training, a random entry was chosen from the set of the current map source. Both the input map and target map were read using *GEMMI* (15), from these maps, a random cube of shape (32, 32, 32) was obtained by interpolating at a random rotation and random translation. During the first 200 epochs of training, the random cube was restricted to cubes containing at least one target grid point in the target map, if the random selection yielded an empty target cube, another was chosen. After 200 epochs, this restriction was removed and any random cube was allowed to be fed to the network for training to emulate better what would be encountered during inference.

#### Infrastructure

The network was trained using the TensorFlow library in Python. The network was trained for 1000 epochs with 1000 steps per epoch and 8 samples per batch. Training was run on a single NVIDIA A40 GPU and took approximately 60 h for each of the three models. The sigmoid focal cross-entropy loss function (16) and Adam optimiser were used for all three networks. To optimise the training speed, the majority of the dataset preparation was precomputed to minimise the sample generation time between batches. In addition, the TensorBoard package was used to optimally match the floating point calculations to the hardware available.

### Inference

When segmenting the electron density of a new nucleic acid-containing structure, a similar workflow is completed to that of the network training data. Namely, a section of the map covering the asymmetric unit is interpolated onto an orthogonal grid with grid spacing 0.7 Å before being split into chunks of shape (32, 32, 32) with an overlap of 16 points between consecutive boxes. A margin consistent with the amount of overlap was added to the asymmetric unit so that the network predicts at each point more than once. Each chunk is then fed to the network and the output of the final layer of the neural network can be trivially converted to a classification using an argmax function. The predictions for each chunk are then reassembled back into the same shape as the interpolated orthogonal grid before being re-interpolated into the original asymmetric unit dimensions and spacing using trilinear interpolation. If using the argmax function for classification, each point in the output of each predicted chunk will have a value of 0 or 1, but averaging overlapping regions during reassembly back to the orthogonal grid and interpolation onto the original asymmetric unit leads to values between 0 and 1 in the output prediction maps.

### Uncertainty estimation

To infer structural information from the predictions outputted by *NucleoFind*, it is often useful to have a measure of uncertainty of a given prediction. *NucleoFind* provides a statistical approach to highlight areas of disagreement between multiple predictions, as well as a direct approach using the output of the deep learning models to highlight low-confidence areas. The statistical approach relies on the fact that *NucleoFind* predicts at each point in the asymmetric unit multiple times, allowing for a point-wise variance to be calculated. This method can be initiated with the ‘-variance’ flag through the command line interface. The output map contains the same dimensions as a standard predicted map, but where every point represents the variance of all of the predictions of that point. Areas with high variance indicate more disagreement between predictions, which could indicate lower confidence. The alternate approach to highlight low-confidence predictions is based on using the raw predicted values output from the deep-learning model. The standard inference procedure uses an argmax function to set predictions greater or equal to 0.5 to the value 1 and points less than 0.5 to the value 0. With the ‘-raw’ command line flag, the argmax function is skipped and a map is generated using the actual positive (’yes’) predicted values in the range 0 to 1. Again, the output map has the same dimensions as a standard predicted map, but where areas with low absolute map values indicate areas of lower confidence.

### Model building

Following the generation of a predicted phosphate feature map, a molecular model can often be built. First, a single, central point for each of the predicted phosphate areas must be obtained for use as the starting estimated phosphorous atom position. All predicted map grid points which are above a threshold value of 0.1 are highlighted. A gradient ascent algorithm is then used to move highlighted grid points to the local maxima predicted map value. From a set of local maxima points, a single position is obtained by averaging all local maxima points with other local maxima points within a 1.5 Å radius. This method produces a single point within each of the predicted phosphate feature map areas, this point is then refined to the local maxima of the 2*mF*_*o*_ − *DF*_*c*_ density whilst remaining within the positive predicted region in the phosphate feature map.

After obtaining a set of estimated phosphorous points from the predicted phosphate map, candidate triplets for library fragment superposition are located. Candidate triplets are defined as three estimated phosphorous points which have a maximum inter-point distance of 8 Å and an angle of 150^○^ ± 50^○^. Trinucleotide fragments from a library structure (PDB Code: 1HR2 (17)) are then superimposed over each candidate triplet in both the 3’ and 5’ directions. The best-fitting superimposed trinucleotide is chosen for each direction, where the sum of the 2mFo – DFc map at the atomic positions of the phosphate and sugar is used to assess the fit. No refinement is performed to optimise the rotation and translation of the fragment as it was not found to provide any benefit over simple superposition over the predicted phosphate positions. Consecutive trinucleotides are grouped into chains, both 3’-5’ and 5’-3’ chains are assessed and the best-fitting chain is accepted, using the average 2mFo – DFc at the atomic positions. If either the sugar or base predicted maps are available, they can optionally be used to score the candidate trinucleotides using the formula presented in Supplementary Equation 1. This may help in the unlikely case that the predicted phosphate map has produced three false-positive predictions with realistic distances and angles to each other. These candidate chains are then grown and processed by the existing algorithms within *Nautilus* (3). The current nucleic acid model-building program was replaced with this new model-building method in the automated model-building pipeline *ModelCraft* version 5.0.0 (18), which is scheduled to be incorporated into the CCP4 software suite (19).

### *ModelCraft* improvements

Small alterations to the *ModelCraft* pipeline were necessary to fully utilise the increased performance of *NucleoFind* over *Nautilus*. The current pipeline performs automated protein model-building with *Buccaneer* (1) before running nucleic acid model-building with *Nautilus*. This scheme was altered to run nucleic acid model building with *NucleoFind* and protein building with *Buccaneer* with the same input map and model. The protein regions of the *Buccaneer* model and the nucleic acid regions of the *NucleoFind* model are then combined into a single model before refinement. Any clashing regions between the two models were solved by removing the highest average scoring region using Equation (1). A clashing region was defined as a collection of contiguous nucleic acid residues which were all within 1 Å of a collection of contiguous protein residues. After this, isolated fragments are removed, defined as residues with no neighbouring atoms within 2.5 Å of bonding atoms.


(1) \begin{eqnarray*} Score = -\frac{1}{n}{\sum _{i}\sum _{j}\sum _{j}{\vert \rho (ijk)\vert }} \end{eqnarray*}

where:

ρ is *mFo* − *DFc* density with a grid spacing of 1 Å
*ijk*is within 5 Å of the clashing residue
*n*is the number of grid points within 5 Å of the clashing residue

### Molecular replacement test set

A test set was generated to test the performance of *NucleoFind* as a tool for building nucleic acids in realistic molecular replacement examples. To generate this test dataset, 1000 protein–nucleic acid X-ray structures that were not part of the training set were randomly selected from the PDB. The structure factors and sequences for this set of structures were downloaded and *MrParse* (22) was run to search the AlphaFold Structural Database for search models (23). Molecular replacement was run on the highest scoring models from *MrParse* using *Slice’N’Dice* (24). The molecular replacement solutions were filtered to a minimum protein completeness of 50 % (i.e. the MR structure has to make up at least 50% of the deposited structure), yielding 288 molecular replacement examples in the test set with a resolution range of 1.35–4.00 Å, shown in Supplementary Figure S5.

## Results and discussion

### Predictions evaluation

**Figure 3.**
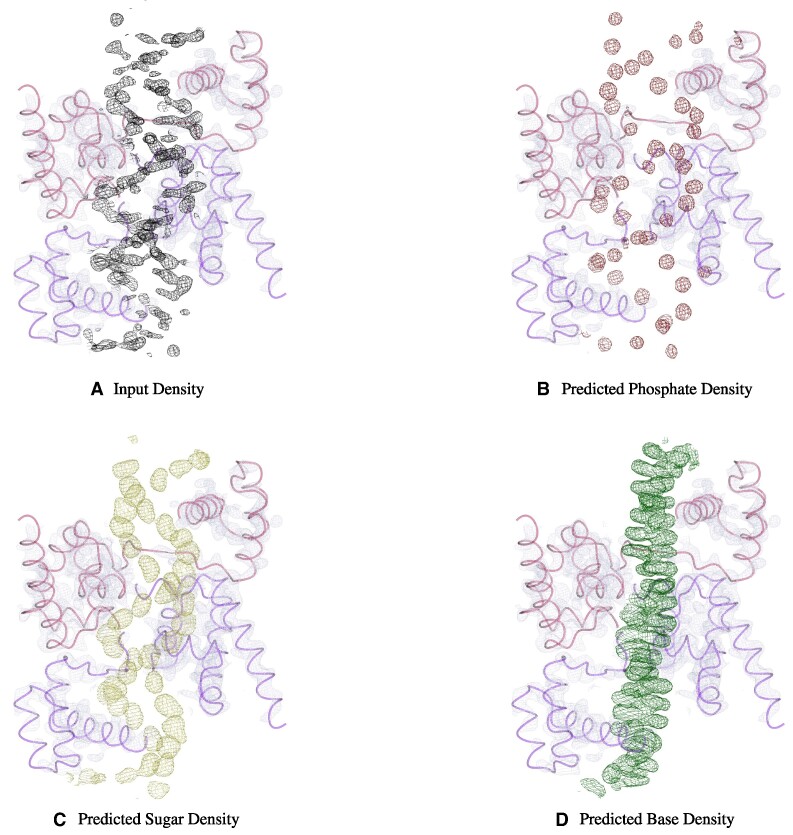
Output of all three deep-learning models corresponding to phosphate group, sugar group and base group predictions. To generate the input density, molecular replacement was performed on a POU DNA binding domain (PDB Code: 3L1P (20) with data to 2.8 Å resolution) using 1HFO (21) as a molecular replacement model. The placed model was refined with *REFMAC5* with a free-R factor of 0.469. The input density, 2mFo – DFc shown in black at 1.5 σ, can be seen as the characteristic DNA duplex. However, the density is noisy and discontinuous which often causes automated model-building software packages to struggle with locating features. *NucleoFind* can highlight the phosphate, sugar and base positions well from the input density, highlighting the usefulness of the program as a post-molecular replacement tool.

The predictions of all three networks of *NucleoFind* are shown in Figure 3, from an input map that was generated from protein molecular replacement. The predicted phosphate map clearly segments the phosphate groups well and the DNA major and minor grooves are evident in the outputted maps. Similarly, the predicted sugar map effectively segments the density corresponding to the sugar ring. Both the predicted phosphate and sugar maps predict regions where the input density is poorly defined or noisy. Interpreting poor-density regions can often be a difficult task for automated software packages, so the ability of *NucleoFind* to provide critical context in these regions is helpful, especially when attempting to build models into maps after protein molecular replacement, as the nucleic acid regions of density are often poorly defined. The base regions of density are most often difficult to observe following protein molecular replacement, yet *NucleoFind* is still able to predict the regions of base density well but with less segmentation between the stacked base-pairs.

To ensure that these trends are consistent across a range of examples, metrics were calculated for all maps predicted from a test set of 288 protein molecular replacement examples which were randomly selected from the PDB and not included in the deep-learning network training, with run times shown in Supplementary Figures S3 and S4. The accuracy of the networks evaluated to 98% or over in all models, not because the networks are almost perfectly accurate, but because the data samples predicted are highly imbalanced. The majority of space sampled corresponds to areas where none of the target groups are present, thus, statistics can become misleading. Furthermore, statistics which only consider the relatively small prediction area (32, 32, 32) may fail to properly encompass the accuracy of the entire predictive map this software provides. Thus, the evaluative metrics shown in Table 1 are calculated using the output predicted maps, which encompass the entire predictive workflow. Further statistics are available in Supplementary Information Section 1.2. To calculate these metrics, all points in the asymmetric unit of the predicted map within 1.5 Å of the target group were labelled. Each labelled point was then compared to that of the predicted map at the same location and marked as either a true positive, true negative, false positive or false negative. Positive predictions were taken as any grid point with a value >0.

**Table 1.** Metrics for the network calculated as an average from a test set of 288 real molecular replacement solution maps

Model	Atom Inclusion (%)	Accuracy (%)	Precision (%)	Recall (%)	F1 score (%)
**Phosphate**	77.6 ± 23.7	99.3 ± 0.4	42.3 ± 10.6	72.3 ± 24.9	52.2 ± 14.9
**Sugar**	85.1 ± 18.6	98.2 ± 1.1	54.1 ± 11.7	81.2 ± 19.6	64.1 ± 14.3
**Base**	83.1 ± 19.2	98.4 ± 1.1	63.7 ± 13.7	84.0 ± 20.1	71.6 ± 15.7

Uncertainty here is represented as the standard deviation across the samples.

The statistics of the network are largely acceptable, with all networks exhibiting good recall and satisfactory precision scores. Achieving both high recall and high precision is an inherent difficulty when predicting such a large number of data points (32^3^) per prediction, and a trade-off is often seen between precision and recall. Nevertheless, it is important to remember the purpose of the network, to segment and interpret electron density, therefore a more useful metric would be to calculate how many of the target groups were within the correctly segmented density. This ‘atom inclusion’ score was simply calculated as the percentage of atoms which were positioned in positive predicted density. The ability of all three models to predict on average 77% of the target atoms or greater with maps generated from molecular replacement is very promising. This, coupled with a good recall score for all of the models suggests that these maps will be very helpful for model building after molecular replacement.

#### Resolution dependence

**Figure 4.**
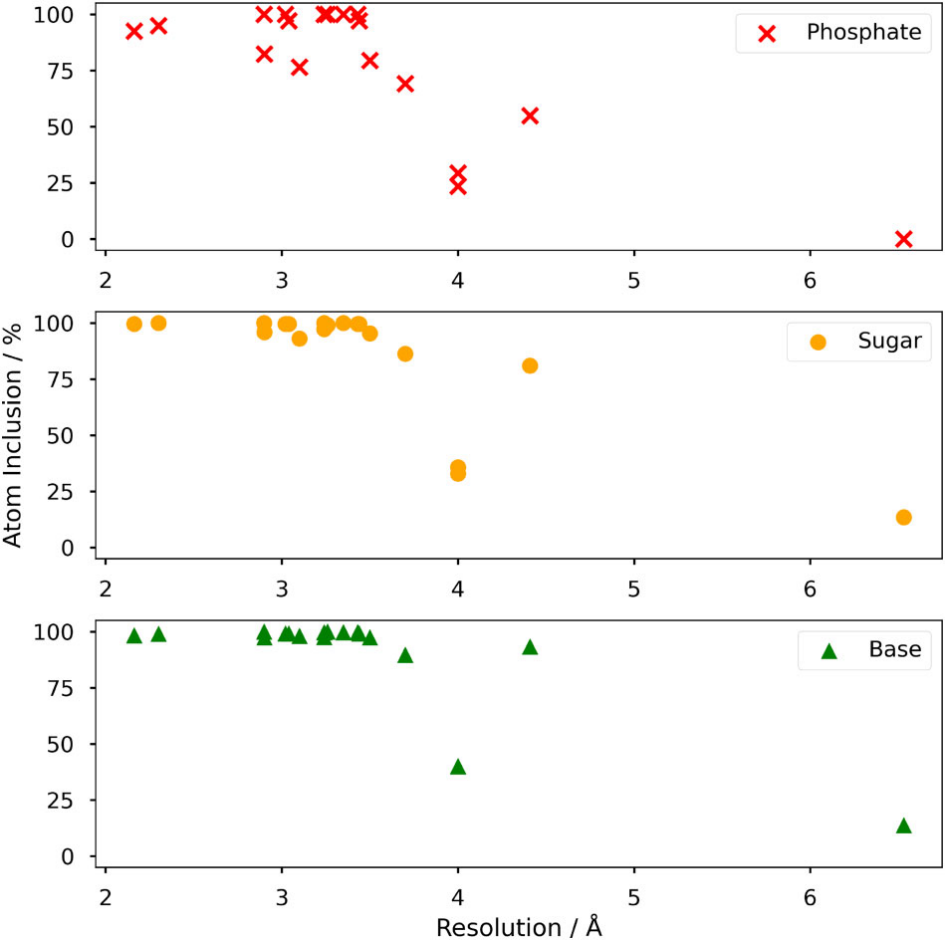
Atom inclusion scores of 20 *NucleoFind* predictions of DNA-bound DNA topoisomerase structures deposited in the PDB with resolutions from 2.11 to 6.35 Å. Maps fed to *NucleoFind* were calculated using only the protein portion of the protein-nucleic acid complex to emulate molecular replacement. *NucleoFind* is able to predict well even at low resolutions.

To test the resolution dependence of *NucleoFind*, predictions were run on twenty DNA-bound DNA topoisomerases proteins in the PDB which have been resolved at varying resolutions between 2.11 and 6.35 Å. Maps were calculated using only the protein domains of the protein-DNA complex to emulate a post-molecular replacement starting map. *NucleoFind* performed well in this test predicting the majority of phosphate, sugar and base atomic positions correctly in structures with resolutions better than 4 Å, shown in Figure 4. Interestingly, the atom inclusion scores of the predicted base and sugar positions tend to be higher for any given structure. This trend is slightly counterintuitive as the more electron-dense phosphorous atoms are often observed to have the strongest features in an electron density map, however the strong atom inclusion scores even at very low resolution for the sugar and base predictions demonstrate the ability of *NucleoFind* to be useful throughout a range of resolutions.

### *De novo* nucleic acid building post molecular replacement

Molecular replacement is a critical method used to obtain phase estimates during structure solution of nucleic acid-containing macromolecules. In particular, this method is particularly useful when attempting to phase protein-nucleic acid complexes as homologous protein models can be obtained readily in many cases, from databases or *in-silico* predictions. After successful molecular replacement, running automated model-building tools can be an efficient way to model the structure of the nucleic acid region. However, the current generation of nucleic acid-building methods in crystallography often struggles with poorly phased data resulting in noisy, hard-to-interpret regions of density. In contrast, *NucleoFind* is often able to provide insightful context describing likely nucleic acid features. The predicted phosphate positions are the most useful for model building since they are most often well resolved with respect to each other, allowing for the nucleic acid topology to be observed. Since the base and sugar groups are larger, the predicted density can become hard to separate, which can cause issues with pinpointing a distinct sugar or base group as a starting point for model building, shown in Figure 3. Nevertheless, the base and sugar groups are useful when determining the likelihood that built fragments are correct.

**Figure 5.**
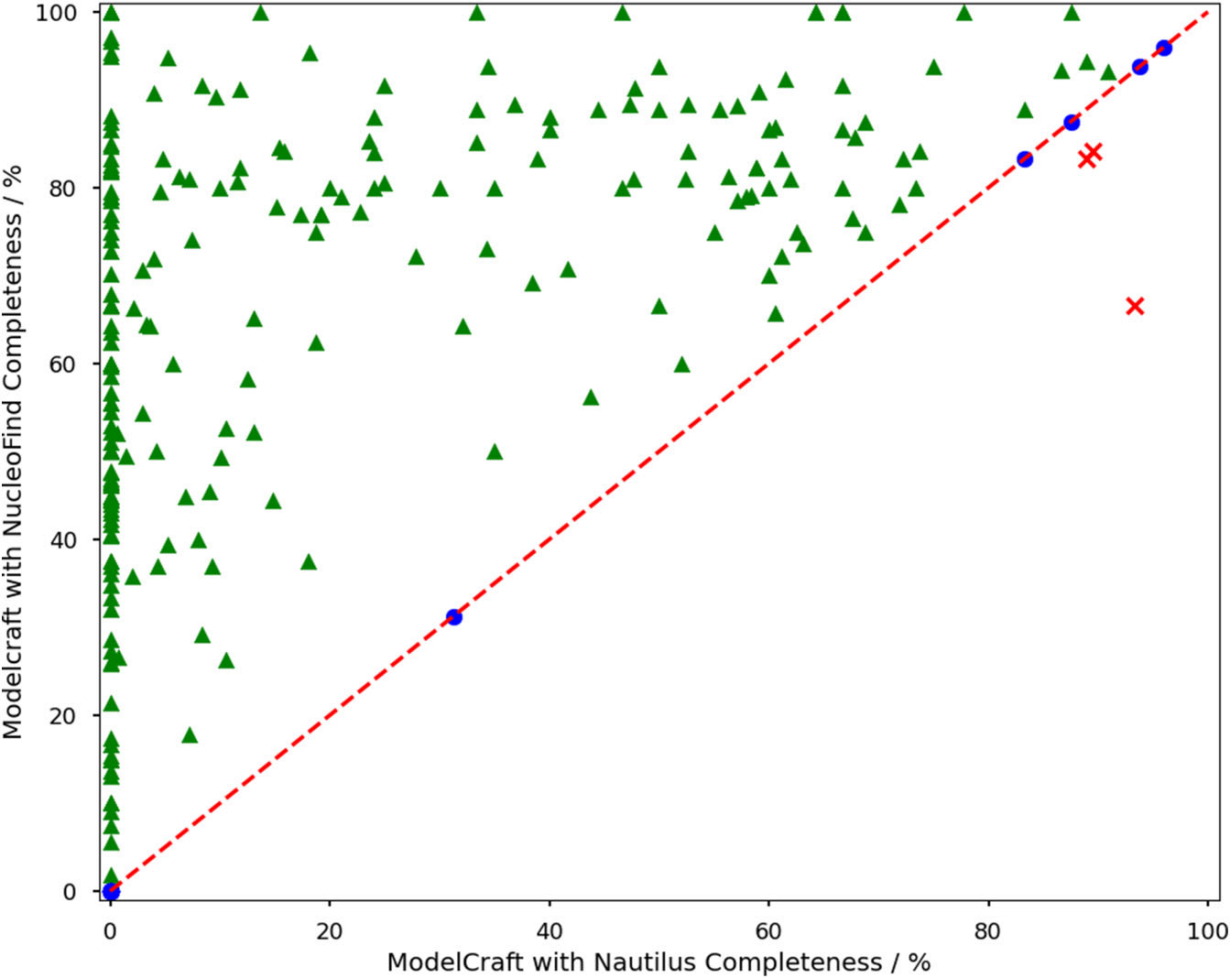
Nucleic acid completeness after five cycles of *ModelCraft* with *NucleoFind* (version 5.0.0) against five cycles of *ModelCraft* with *Nautilus* (version 3.3.0), both with default parameters. ModelCraft version 3.3.0 contains *Nautilus* as the only nucleic acid model building software which runs after protein model building with *Buccaneer*. ModelCraft version 5.0.0 contains *NucleoFind* as the only nucleic acid model building software which is run alongside protein model building with *Buccaneer* and the results combined. The 288 protein–nucleic structures used in this test were generated by protein molecular replacement.

A test of 288 molecular replacement examples showed improved automated model-building performance compared to the current generation of methods in the vast majority of cases, the completeness after five cycles of *ModelCraft* with default parameters is shown in Figure 5. On average, the new version of *ModelCraft* with *NucleoFind* built 61.1% of residues within 1.5 Å of the deposited model, whereas *ModelCraft* with *Nautilus* was only able to build 20.7% of residues on average. *NucleoFind* has the ability to build a large portion of the unmodelled nucleic acids into realistic density in seconds, allowing for better phase estimates to be obtained with little input from the user. Incorporating this new tool into *ModelCraft* can allow for high-throughput structure solution, as may be more commonly required in the future with developments in methods such as serial crystallography.

#### Case study 1: de novo building of Thermus thermophilus 30S ribosomal subunit

Understanding ribosomal structure has provided crucial insight into the biochemistry underpinning protein synthesis (25). One of the many challenges faced when attempting to solve the structure of such large complexes is obtaining sufficiently good phase estimates. Multiple experimental phasing strategies were employed when solving ribosomal subunits which primarily relied on heavy atom soaking (26). To test the ability of *NucleoFind* to build ribosomal RNA after protein molecular replacement, the structure solution process of the 30S ribosome was repeated, starting with 3.37 Å resolution merged reflection data from PDB entry 1IBK (27).

**Figure 6.**
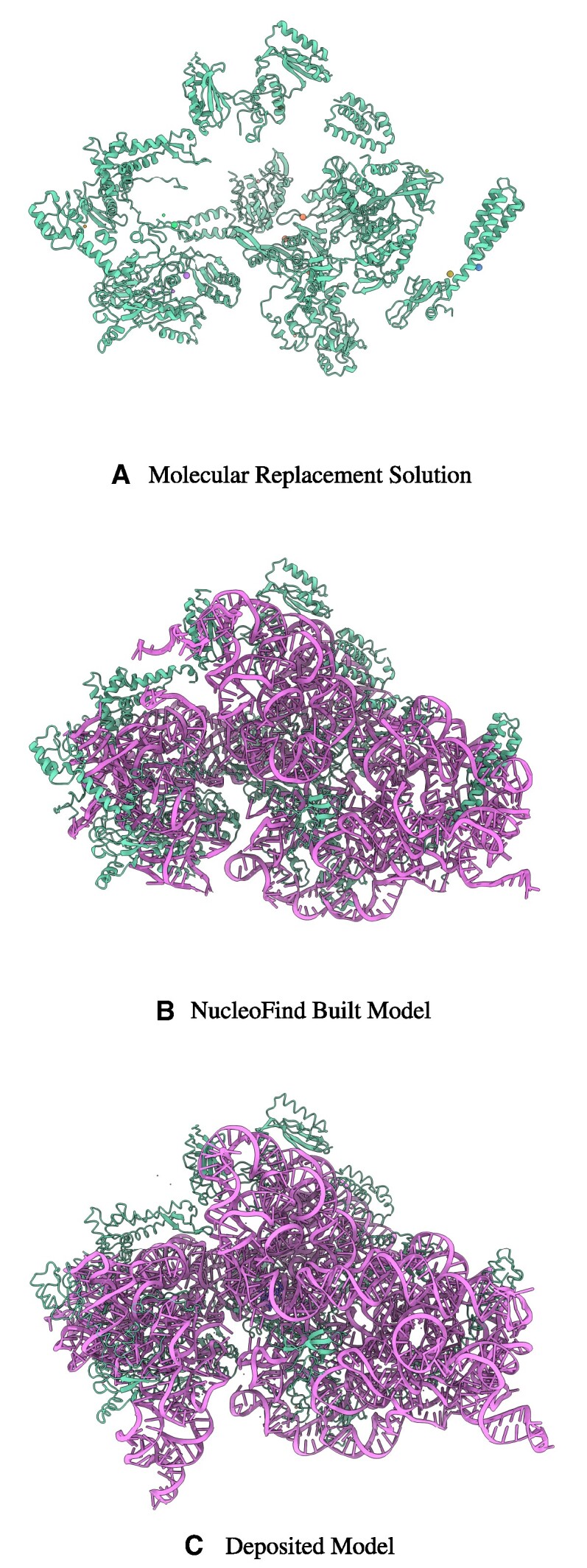
(**A**) Protein only molecular replacement solution solved with *Phaser* (PDB Code: 2PQE). (**B**) Model built with automated model building software package *ModelCraft* with *NucleoFind*. A large proportion of the model is built from the molecular replacement starting point in a short amount of time. (**C**) Deposited model (PDB Code: 1IBK). The green colour represents protein and the purple colour represents nucleic acid. Additional images are shown in Supplementary Figure S6.

A homologous protein model, 2PQE (28), was found using *MrBump* (29), and molecular replacement was performed with *Phaser* (30). The molecular replacement model was then run through 5 cycles of *ModelCraft* which contained *NucleoFind* as the only nucleic acid model builder with default parameters. In this test, *ModelCraft* with *NucleoFind* produces an outstanding result, building 1014 sugar-phosphate groups out of the 1525 nucleic acids in the deposited model with a maximum atomic position deviation of 1.5 Å, shown in Figure 6. The previous generation method, *ModelCraft* with *Nautilus* struggles, building only 119 sugar-phosphate groups within 1.5 Å of the deposited model. The order of magnitude improvement between the two methods is primarily due to the deep-learning prediction aiding the location of potential phosphate group sites. The model output by *ModelCraft* with *NucleoFind* provides a substantially improved phase estimate and model-building starting point in just three hours of automated computation time with commodity hardware, with each run of *NucleoFind* taking only ten minutes. The regions where *NucleoFind* was unable to confidently build were areas of weaker density far removed from protein, it is likely that *NucleoFind* could build into this area provided relaxation of the building cutoff thresholds, however, by default these cutoffs are quite strict to ensure any fragments built are of relatively high confidence.

#### Case study 2: de novo building of CRISPR-–Cas12c1 DNA–RNA ternary complex after AlphaFold 3 prediction

The CRISPR–Cas12c1 system is an example of a complex protein-nucleic acid system which is useful for guided genome editing. The Cas12 protein is able to bind two forms of nucleic acid, a single guard RNA (sgRNA) and a target double-stranded DNA (dsDNA) (31). Structural insight of the complex is imperative to convert this system into an efficient and effective gene editing tool. The structure was solved at 3.20 Å resolution using single-wavelength anomalous diffraction (SAD) with a selenomethionine derivative (PDB Code: 7VYX).

Predictive tools like AlphaFold 3 (32) and RosettaFold2NA (33) aim to produce predictive models of difficult protein-nucleic complexes. If a sufficiently good prediction can be obtained, these models can be used wholly or in parts in molecular replacement, however in many cases accurate predictions of protein-RNA/DNA complexes remain challenging. Performing molecular replacement with erroneous predicted models often leads to difficulty finding a solution, however taking only the confident regions of a prediction can be a viable strategy to obtain a modelling starting point from which automated model building may be able to progress the model further. To test this, AlphaFold 3 was used to predict the structure of the CRISPR–Cas12c1 system in complex with sgRNA and dsDNA. The AlphaFold 3 predicted model contains 1310 amino acid residues with an average pLDDT of 78.5 and 190 nucleic acid residues with an average pLDDT of 35.0. 89.3% of the amino acid residues have a pLDDT >60, whereas only 15.2% of nucleic residues are above the same level. Attempting molecular replacement with the full AlphaFold 3 model does not yield a molecular replacement solution with *Phaser*, however, trimming the model to contain residues with a pLDDT >60 allows a solution with an R-free value of 0.504 to be found. 200 cycles of jelly-body refinement with *Refmacat* (34) reduces the *R*-free value to 0.43. The resulting model after 10 cycles of *ModelCraft* with *NucleoFind* built 49.6 % of the nucleic acid residues, a 39.3 percentage point increase over the resulting model after 10 cycles of *ModelCraft* with *Nautilus* (version 3.3.0). The majority of the dsDNA and parts of the sgRNA chains are well-modelled which provides a substantially better starting point than previous versions of *ModelCraft*. Images are shown in Supplementary Figure S7.

## Conclusions

In conclusion, *NucleoFind* is able to interpret and segment nucleic acid electron density. It produces feature maps identifying three key components of nucleotide structure. These feature maps provide exceptional context for automated nucleic acid model-building software, and *NucleoFind* can utilise this for significant improvements in model-building capability. This work has only reported examples of protein-nucleic acid complexes solved by protein molecular replacement, but we expect *NucleoFind* to also give an improvement after experimental phasing or molecular replacement with a partial nucleic-acid structure. In addition to automated model building, the predicted maps generated by *NucleoFind* can be used as a guide for interactive nucleic acid model building, which may particularly benefit less experienced users.

One of the features identified by this software is the ribose ring of the nucleotide backbone. Another future application of this approach will be the adaptation of this feature to the determination of carbohydrate ligands and modifications. The software is openly available for reuse in other packages and can be routinely installed as a Python package. We welcome its use in other software pipelines in whatever form is useful.

## Supplementary Material

gkae715_Supplemental_File

## Data Availability

All code used to generate datasets, train deep-learning networks and perform model inference are available on Zenodo (10.5281/zenodo.12527875). The released version of NucleoFind is available at https://github.com/Dialpuri/NucleoFind.
