# Supplementary material for: NucleoFind: a deep-learning network for interpreting nucleic acid electron density
Source: Nucleic Acids Res. 2024 Aug 20;52(17):e84. doi: 10.1093/nar/gkae715 (PMC11417358; doi:10.1093/nar/gkae715)
Supplement: gkae715_Supplemental_File [file gkae715_supplemental_file.pdf]

NucleoFind: A Deep-Learning Network for Interpreting Nucleic Acid Electron Density Supplementary Information

Jordan S. Dialpuri, Jon Agirre, Kathryn D. Cowtan and Paul S. Bond

2024

1 Neural Network

1.1 Network Architecture

Supplementary Algorithm 1 3D U-Net

$Input \leftarrow$ 32x32x32x1
$x \leftarrow conv\_block(filters = 16)$
$x \leftarrow conv\_block(filters = 32)$
$x \leftarrow conv\_block(filters = 64)$
$x \leftarrow conv\_block(filters = 128)$
$x \leftarrow conv\_block(filters = 256)$
$x \leftarrow conv3d(filters = 512, activation = relu, kernel\_initializer = he\_normal)$
$x \leftarrow conv3d(filters = 512, activation = relu, kernel\_initializer = he\_normal)$
$x \leftarrow deconv\_block(filters = 256)$
$x \leftarrow deconv\_block(filters = 128)$
$x \leftarrow deconv\_block(filters = 64)$
$x \leftarrow deconv\_block(filters = 32)$
$x \leftarrow deconv\_block(filters = 16)$
$x \leftarrow conv3d(filters = 2, activation = softmax, padding = same)$
$Output \leftarrow$ 32x32x32x2

Supplementary Algorithm 2 conv_block

$x \leftarrow Conv3d(filters, padding = same, use\_bias = False, strides = 1)$
$x \leftarrow GroupNormalisation()$
$x \leftarrow ReLU()$
$x \leftarrow Conv3d(filters, padding = same, use\_bias = False, strides = 1)$
$x \leftarrow GroupNormalisation()$
$x \leftarrow ReLU()$
$x \leftarrow MaxPool3D(pool\_size = 2)$

Supplementary Algorithm 3 deconv_block

$x \leftarrow Conv3dTranspose(filters, kernel\_size = 3, stride = 2, padding = same)$
$x \leftarrow Concatenate(downsampling\_layer)$
$x \leftarrow Conv3d(filters, kernel\_size = 3, padding = same, kernel\_initializer = he\_normal)$
$x \leftarrow GroupNormalisation()$
$x \leftarrow ReLU()$
$x \leftarrow Conv3d(filters, kernel\_size = 3, padding = same, kernel\_initializer = he\_normal)$
$x \leftarrow GroupNormalisation()$
$x \leftarrow ReLU()$

where GroupNormalisation has parameters

- axis=-1
- center=True
- scale=True
- beta_initializer=random_uniform
- gamma_initializer=random_uniform

1.2 Model Statistics

Full average statistics are listed for all three models from a test set of 288 protein-nucleic acid complex molecular replacement examples:

Supplementary Table 1: Table of model statistics for the three deep-learning networks, ± indicates standard deviation.

Metric	Phosphate	Sugar	Base
True Positives	6506.7 ± 9762.1	25752.8 ± 43834.8	33747.2 ± 56041.5
True Negatives	1643646.5 ± 2358273.5	1605705.4 ± 2288929.2	1600727.0 ± 2279551.1
False Positives	8992.0 ± 14071.8	22957.1 ± 42384.6	20937.7 ± 43274.1
False Negatives	3567.0 ± 8877.0	8297.0 ± 22926.3	7300.4 ± 21220.1
Accuracy	99.3 ± 0.4 %	98.2 ± 1.1 %	98.4 ± 1.1 %
Precision	42.3 ± 10.6 %	54.1 ± 11.7 %	63.7 ± 13.7 %
Recall	72.3 ± 24.9 %	81.2 ± 19.6 %	84.0 ± 20.1 %
F1 Score	52.2 ± 14.9 %	64.1 ± 14.3 %	71.6 ± 15.7 %
False Positive Rate	0.6 ± 0.3 %	1.4 ± 0.7 %	1.2 ± 0.8 %
False Negative Rate	27.7 ± 24.9 %	18.8 ± 19.6 %	16.0 ± 20.1 %
Error	0.7 ± 0.4 %	1.8 ± 1.1 %	1.6 ± 1.1 %
Sensitivity	72.3 ± 24.9 %	81.2 ± 19.6 %	84.0 ± 20.1 %
Specificity	99.4 ± 0.3 %	98.6 ± 0.7 %	98.8 ± 0.8 %

1.3 Training Data

1.3.1 Resolution

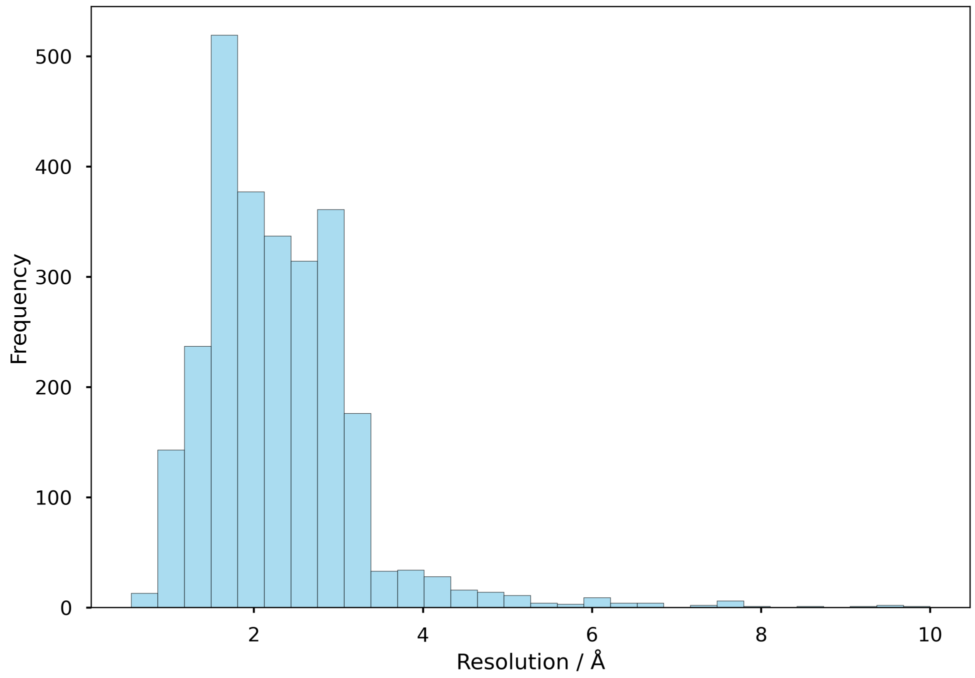

Supplementary Figure 1: Histogram of resolution of nucleic acid only structures used in the dataset before test-train split

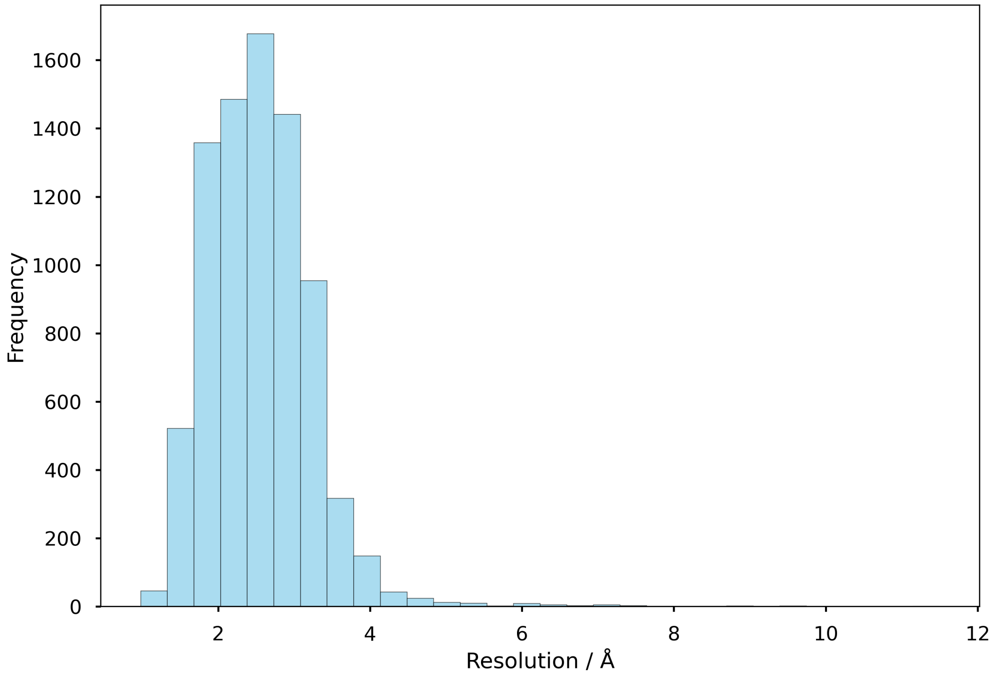

Supplementary Figure 2: Histogram of resolution of protein-nucleic acid structures used in the dataset before test-train split

1.4 Inference

1.4.1 Run Time

The run times of 288 molecular replacement examples are shown in Figure 3 against unit cell volume and in Figure 4 against asymmetric unit volume.

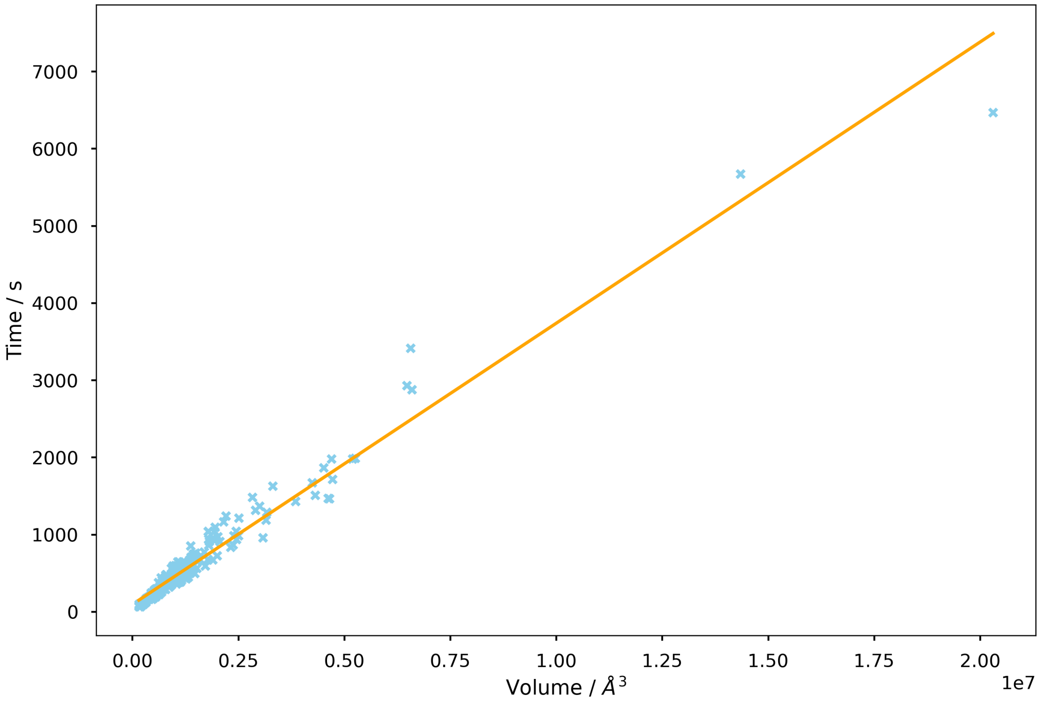

Supplementary Figure 3: Run times of 288 molecular replacement structures against unit cell volume when predicting over the entire unit cell.

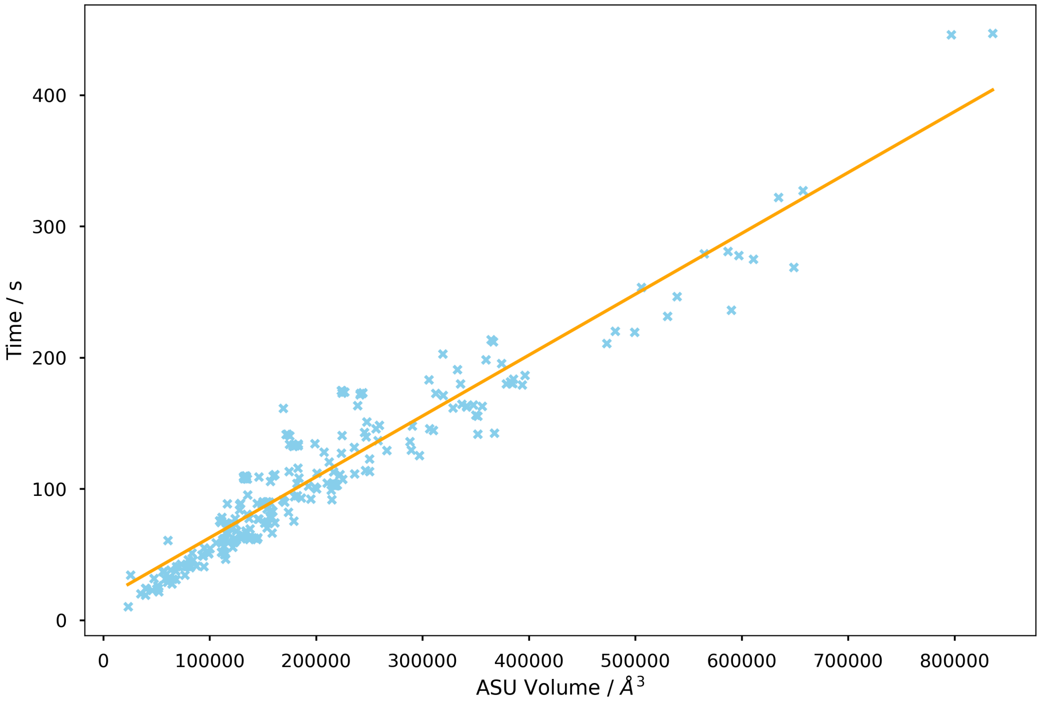

Supplementary Figure 4: Run times of 288 molecular replacement structures against asymmetric unit volume when predicting over only the asymmetric unit.

2 Model Building

2.1 Scoring

The scoring algorithm used when assessing the fit of residue into the experimental density with optional additional scoring from the predicted sugar and predicted base maps, is shown in Equation 1.

$$Score = \sum_{i=0}^{n} \rho_e(a_i) + \sum_{i=0}^{n_{sugar}} \rho_s(a_i) + \sum_{i=0}^{n_{base}} \rho_b(a_i) \tag{1}$$

where:

ρ_e is the $2mFo - DFc$ (experimental) map

ρ_s is the predicted sugar map

ρ_b is the predicted base map

a_i is the ith atom in a set of atoms

n is a set of all non-hydrogen atoms in the phosphate group, sugar group and the ring-attached nitrogen of the base group of a given residue.

n_{sugar} is a set of all non-hydrogen atoms in the sugar group

n_{base} is a set of all non-hydrogen atoms in the base group

2.2 Molecular Replacement Test Set

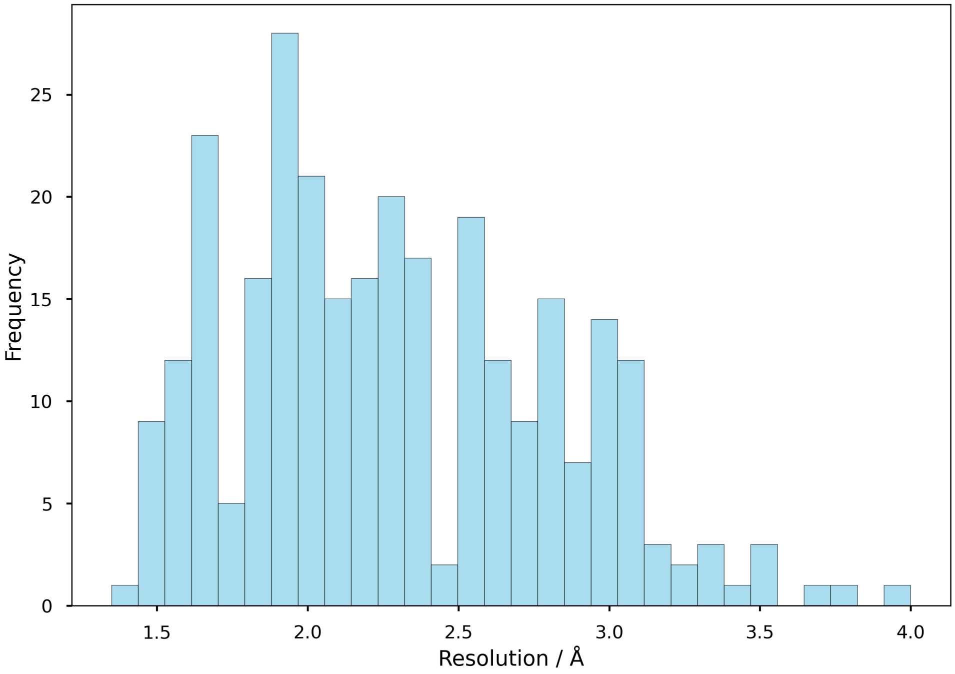

Supplementary Figure 5: Histogram of resolutions of 288 molecular replacement structures used as a model building test set.

2.3 Case Study 1: De novo building of *Thermus Thermophilus* 30S ribosomal subunit

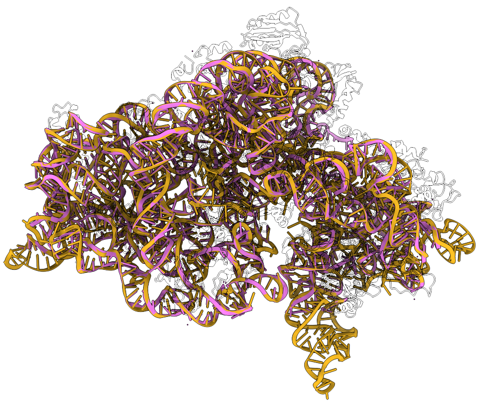

(a) Front View

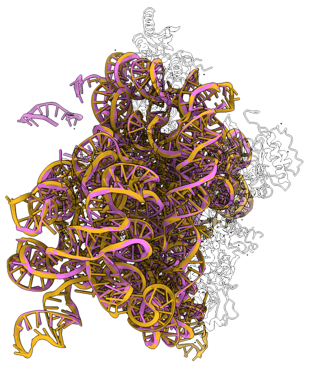

(b) Side View

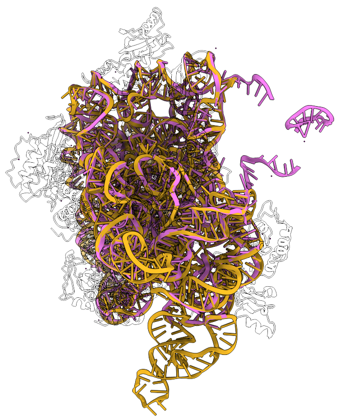

(c) Side View

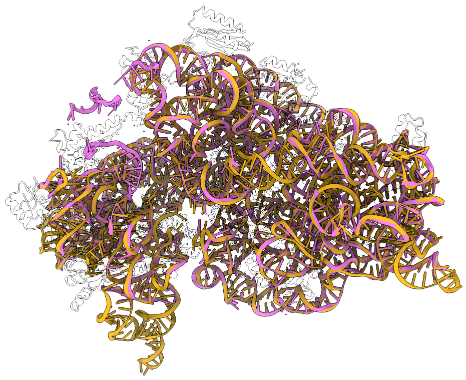

(d) Back View

Supplementary Figure 6: Deposited *Thermus Thermophilus* 30S ribosomal subunit (protein - outlined, nucleic acid - gold), superposed with the model built with automated model building software package *ModelCraft* with *NucleoFind* (nucleic acid - pink) *ModelCraft* with *NucleoFind* builds well in almost all areas. The largest area of difference is a looped nucleic acid region shown at the bottom of both the front and back figures, which is present in the deposited model but not in the built model.

2.4 Case Study 2: De novo building of CRISPR-Cas12c1 DNA-RNA ternary complex after AlphaFold 3 prediction

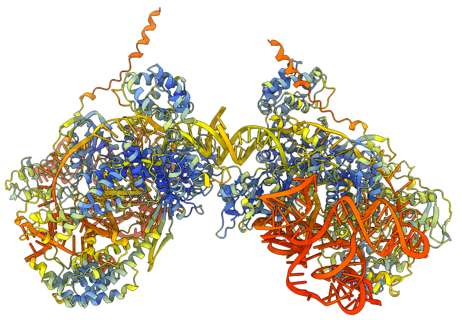

(a) AlphaFold 3 Model

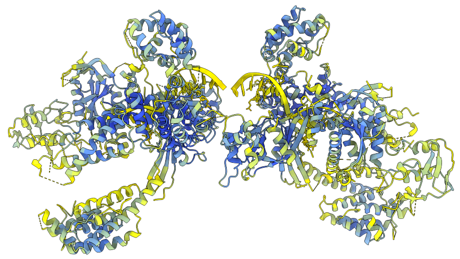

(b) Trimmed AlphaFold 3 Model

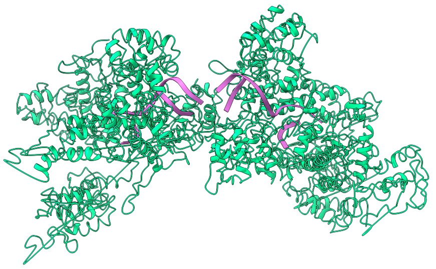

(c) ModelCraft 3.3.0 Model

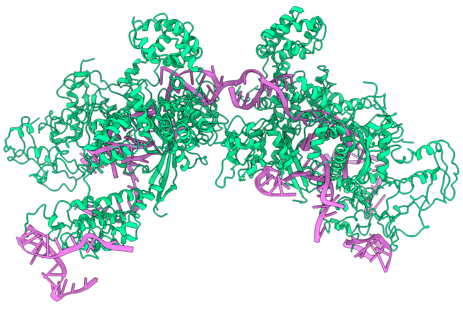

(d) ModelCraft 5.0.0 Model

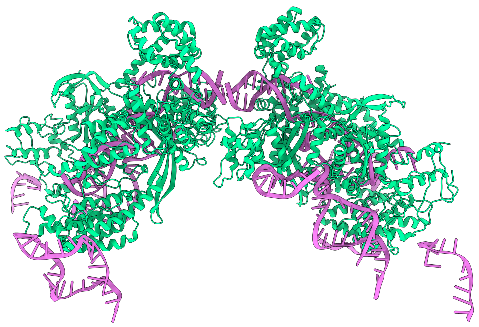

(e) Deposited Model

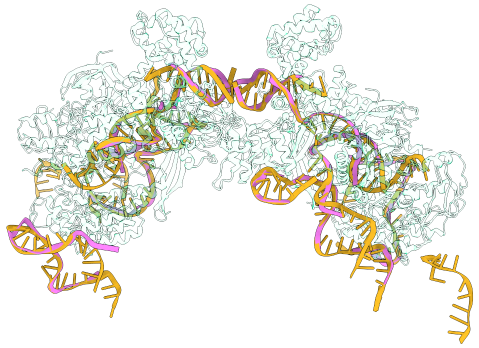

(f) Superimposed Model

Supplementary Figure 7: A - Image of AlphaFold 3 model of CRISPR-Cas12c1 DNA-RNA complex, coloured by pLDDT. B - Image of AlphaFold 3 model of CRISPR-Cas12c1 DNA-RNA complex with residues with pLDDT less than 60 removed. C - Output of ModelCraft version 3.3.0 after 10 cycles. D - Output of ModelCraft version 5.0.0 after 10 cycles showing more accurate nucleic acid model building. E - Deposited model (PDB Code: 7VYX). F - Superposition of deposited model (nucleic acid - gold) and ModelCraft 5.0.0 model (nucleic acid - pink).
